# Magnetic CoFe_1.95_Y_0.05_O_4_-Decorated Ag_3_PO_4_ as Superior and Recyclable Photocatalyst for Dye Degradation

**DOI:** 10.3390/ma16134659

**Published:** 2023-06-28

**Authors:** Qingwang Liu, Mai Xu, Ying Meng, Shikun Chen, Shiliu Yang

**Affiliations:** School of Chemistry and Materials Engineering, New Energy Materials and Technology Research Center, Huainan Research Center of New Carbon Energy Materials, Anhui Key Laboratory of Low Temperature Co-Fired Materials, Huainan Normal University, Huainan 232038, China

**Keywords:** Ag_3_PO_4_/CoFe_1.95_Y_0.05_O_4_, photocatalytic degradation, methylene blue, rhodamine B, magnetic separation

## Abstract

The Ag_3_PO_4_/CoFe_1.95_Y_0.05_O_4_ nanocomposite with magnetic properties was simply synthesized by the hydrothermal method. The structure and morphology of the prepared material were characterized, and its photocatalytic activity for degradation of the methylene blue and rhodamine B dyes was also tested. It was revealed that the Ag_3_PO_4_ in the nanocomposite exhibited a smaller size and higher efficiency in degrading dyes than the individually synthesized Ag_3_PO_4_ when exposed to light. Furthermore, the magnetic properties of CoFe_1.95_Y_0.05_O_4_ enabled the nanocomposite to possess magnetic separation capabilities. The stable crystal structure and effective degradation ability of the nanocomposite were demonstrated through cyclic degradation experiments. It was shown that Ag_3_PO_4_/CoFe_1.95_Y_0.05_O_4_–0.2 could deliver the highest activity and stability in degrading the dyes, and 98% of the dyes could be reduced within 30 min. Additionally, the photocatalytic enhancement mechanism and cyclic degradation stability of the magnetic nanocomposites were also proposed.

## 1. Introduction

The main hazardous substances in wastewater come from chemical dyes, which can pollute the environment, destroy the ecological balance, and increase the risk of cancer in humans. Therefore, it is extremely urgent to develop efficient wastewater treatment technologies to degrade these chemical dyes. Semiconductor photocatalytic oxidation has been widely considered as one of the most promising photocatalytic technologies to achieve this goal [1,2,3,4,5]. Traditional TiO_2_- or ZnO-based materials have been studied for a long time as photocatalysts due to their exceptional merits of high electron mobility, high stability, non-toxicity, and low cost. However, both substances have relatively high band gap energies that can only be activated by UV irradiation, limiting their potential applications within the wavelength range of sunlight [6,7,8,9].

Therefore, many efforts have been made to explore new photocatalysts with narrow band gaps under certain conditions to achieve a higher light energy conversion efficiency [10,11,12,13]. One of the most promising photocatalysts is silver phosphate (Ag_3_PO_4_), which has an extremely high photooxidation capacity, a high quantum efficiency of around 90%, and a low band gap of approximately 2.5 eV. As a result, Ag_3_PO_4_ has been extensively studied in degrading organic pollutants in water due to its good photocatalytic activity [14,15,16,17]. Nevertheless, the reported Ag_3_PO_4_ usually has a larger particle size and smaller specific surface area, and has a lower stability with prolonged exposure to light, leading to an unsatisfactory photocatalytic activity and stability. Furthermore, Ag_3_PO_4_ is difficult to recycle, leading to a high cost of the photocatalyst as well as secondary pollution to water resources.

In order to optimize the photocatalytic properties of Ag_3_PO_4_ in practical applications, it is essential to design matrix materials with a smaller particle size and long-term stability. An effective approach to achieve this is to create a heterojunction structure by coupling Ag_3_PO_4_ with another substance [14,16,18]. The heterojunction structure can facilitate the separation of photogenerated electrons and holes, preventing electron–hole recombination and improving the stability of Ag_3_PO_4_. It is reported that cobalt ferrite (CoFe_2_O_4_) can exhibit a high electron transfer capability and stability, plus ferromagnetic properties [19,20,21], Additionally, it is also discovered that the visible-light adsorption of CoFe_2_O_4_ can be further enhanced by rare-earth-metal doping [22,23]. Therefore, through combining the rare-earth-metal-doped CoFe_2_O_4_ with Ag_3_PO_4_, the nanocomposites should present high photocatalytic activity, long degradation stability, and designable magnetic properties.

In this paper, an Ag_3_PO_4/_CoFe_1.95_Y_0.05_O_4_ nanocomposite with magnetic properties was easily synthesized by the hydrothermal method. The structure and morphology of the prepared material were characterized, and its photocatalytic activity was also tested for degradation of the methylene blue and rhodamine B dyes. Especially, the influence of the mass ratio of Ag_3_PO_4_ to CoFe_1.95_Y_0.05_O_4_ on the photocatalytic property was discussed. It was revealed that Ag_3_PO_4_/CoFe_1.95_Y_0.05_O_4_–0.2 exhibited a higher efficiency in degrading dyes than Ag_3_PO_4_ when exposed to light. Additionally, the photocatalytic enhancement mechanism and cyclic degradation stability of the magnetic nanocomposites were also proposed.

## 2. Materials and Methods

### 2.1. Materials

Cobalt nitrate hexahydrate, iron (III) nitrate nonahydrate, citric acid, yttrium nitrate hexahydrate, silver nitrate, polyvinylpyrrolidone, disodium hydrogen phosphate dodecahydrate, methylene blue, and rhodamine B drugs were purchased from Sinopharm Chemical Reagent Co., Ltd. (Shanghai, China); the reagents used were analytically pure; and the solution was prepared with deionized water.

### 2.2. Synthesis of Samples

#### 2.2.1. Preparation of CoFe_1.95_Y_0.05_ O_4_

Typically, 2.850 g of cobalt nitrate hexahydrate (15.6 mmol), 7.429 g of iron (III) nitrate nonahydrate (30.60 mmol), 6.245 g of citrate (32.50 mmol), and 0.018 g of yttrium nitrate hexahydrate (0.05 mmol) were mixed in a 150 mL beaker, then 50 mL of deionized water was poured into the beaker and magnetically stirred at room temperature for one hour to form a transparent solution. Next, 12 mol·L^−^^1^ of ammonia was dropwise-added to the solution until reaching a pH value of 6–7. The solution was evaporated at a constant temperature of 100 °C for 24 h to obtain a dry gel. Afterward, the gel was calcinated at 700 °C for 3 h with a heating rate of 2.5 °C·min^−^^1^. Finally, the sample was cooled to room temperature and ground for future use.

#### 2.2.2. Preparation of Ag_3_PO_4_/CoFe_1.95_Y_0.05_O_4_

Different amounts of the prepared CoFe_1.95_Y_0.05_O_4_ (0.1 g, 0.2 g, and 0.3 g) were ultrasonically dispersed in 100 mL of deionized water. Then, 0.840 g of silver nitrate (4.90 mmol), 1.791 g of disodium hydrogen phosphate dodecahydrate (12.60 mmol), and 1.2 g of polyvinylpyrrolidone were dissolved into the above suspension, which was hydrothermally heated at 200 °C for 24 h. After that, the samples were cooled, filtered, washed by water three times, and dried at 60 °C for 10 h. The samples with different amounts of CoFe_1.95_Y_0.05_O_4_ were named Ag_3_PO_4_/CoFe_1.95_Y_0.05_O_4_–0.1, Ag_3_PO_4_/CoFe_1.95_Y_0.05_O_4_–0.2, and Ag_3_PO_4_/CoFe_1.95_Y_0.05_O_4_–0.3, respectively. The individually synthesized pure Ag_3_PO_4_ was prepared by the same procedure without adding CoFe_1.95_Y_0.05_O_4_.

### 2.3. Analytical Characterization

The samples’ crystal structure was measured by a DX-2800 diffractometer with CuKα (λ = 0.154056 nm) and a scanning range of 2θ = 5°~80°. The morphology and crystalline structure were observed with a ZEISS Sigma500 Scanning Electron Microscope (SEM, Carl Zeiss AG, Oberkochen, Germany) and a FEI Talos-F200S Transmission Electron Microscope (TEM, FEI Company, Hillsboro, USA).The diffuse reflectance spectra were determined with a UV-3700 UV-Vis spectrometer (Shimadzu, Kyoto, Japan). Total organic carbon (TOC) was analyzed using a Shimadzu TOC-5000A analyzer (Shimadzu, Kyoto, Japan), and photocatalytic testing was performed using the ZQ-GHX-V (Shanghai Zhengqiao Scientific Instruments Co., Ltd., Shanghai, China). Electrochemical Impedance Spectroscopy (EIS) was recorded using an electrochemical workstation (CHI660E, CH Instruments, Austin, TX, USA) under a frequency range of 0.1–100 KHz in 0.5 M sodium sulfate solution.

### 2.4. Measurement of Photocatalytic Activity

To achieve adsorption equilibrium between the photocatalyst and the dye, 0.1 g of each catalyst was added to 50 mL of 1.0 × 10^−^^5^ mol·L^−^^1^ solution containing either methylene blue (MB) or rhodamine B (RhB), respectively. The mixture was stirred away from light for one hour. Subsequently, the dye solution was subjected to catalytic degradation evaluation under a 500 W xenon lamp. In the photocatalytic process, approximately 5.0 mL of the solution was extracted every 5 min and its absorbance was measured using a UV-vis spectrometer at the maximum absorption wavelength of MB (665 nm) and RhB (550 nm). Furthermore, the photocatalytic effect of the catalyst on various concentrations of dyes was also examined. The TOC values throughout the degradation process were determined by a Shimadzu TOC-5000A analyzer.

## 3. Results and Discussions

### 3.1. Structure and Morphology

**Figure 1:**
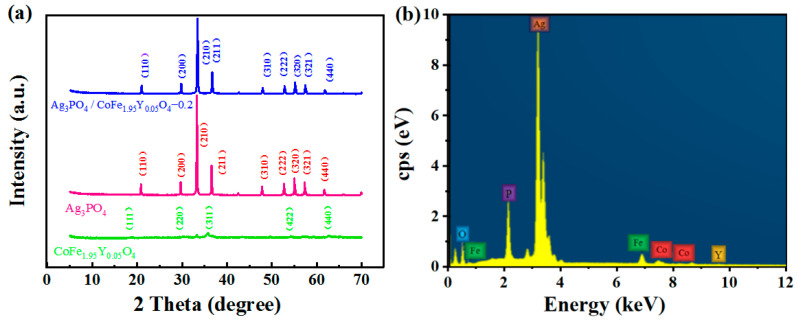
(**a**) XRD patterns of the different samples; (**b**) EDS pattern of the Ag_3_PO_4_/CoFe_1.95_Y_0.05_O_4_–0.2.

The XRD patterns of each sample are displayed in Figure 1a. The main peaks of the pure Ag_3_PO_4_ can be indexed to the standard diffraction of Ag_3_PO_4_ [17] (JCPDS No. 06-0505), while the main peaks of CoFe_1.95_Y_0.05_O_4_ can be indexed to the standard diffraction of CoFe_2_O_4_ (JCPDS No. 22-1086). However, the main peaks of the Ag_3_PO_4_/CoFe_1.95_Y_0.05_O_4_–0.2 composite are all indexed to Ag_3_PO_4_. The proper reason should be that the diffraction peaks of Ag_3_PO_4_ are so strong that the crystallographic planes of CoFe_1.95_Y_0.05_O_4_ are concealed; as shown in Figure 1a, the peak’s intensity of CoFe_1.95_Y_0.05_O_4_ is much lower than that of Ag_3_PO_4_. The Energy-Dispersive X-Ray spectrum (EDS) confirms that Ag_3_PO_4/_CoFe_1.95_Y_0.05_O_4_–0.2 is composed of Co, Fe, Y, O, Ag, and P elements (Figure 1b).

**Figure 2:**
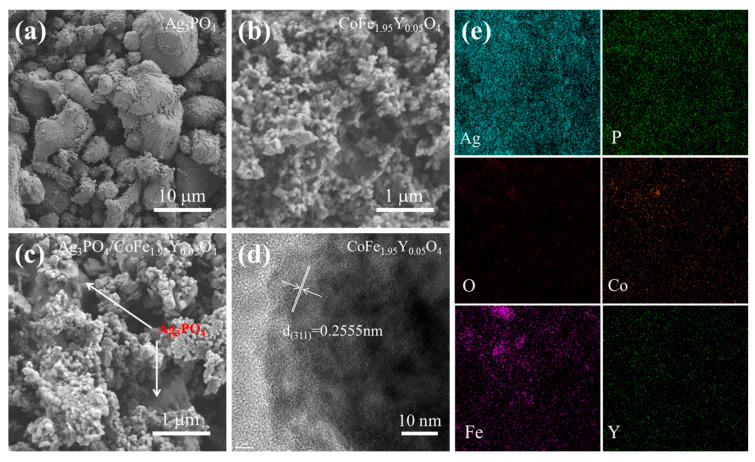
SEM images of (**a**) Ag_3_PO_4_, (**b**) CoFe_1.95_Y_0.05_O_4_, and (**c**) Ag_3_PO_4/_CoFe_1.95_Y_0.05_O_4_–0.2; (**d**) TEM image of the CoFe_1.95_Y_0.05_O_4_ in composite; (**e**) EDS mapping of the Ag_3_PO_4_/CoFe_1.95_Y_0.05_O_4_–0.2.

Figure 2a–c display the SEM images of the pure Ag_3_PO_4_, CoFe_1.95_Y_0.05_O_4_, and Ag_3_PO_4_/CoFe_1.95_Y_0.05_O_4_–0.2 nanocomposites, and it is evident that the individually synthesized Ag_3_PO_4_ has an irregular granular morphology with a large size of approximately 5–10 μm, while the Ag_3_PO_4_ in the composite exhibits a significantly smaller size around 1 μm, demonstrating that the CoFe_1.95_Y_0.05_O_4_ nanoparticles plays an important role in reducing the size of Ag_3_PO_4_. The nanoparticles decorated on the microparticles are CoFe_1.95_Y_0.05_O_4_, because the lattice spacing in the TEM image is indexed to the (311) plane of CoFe_1.95_Y_0.05_O_4_ (Figure 2d). It is difficult to observe the lattice planes of the microsized Ag_3_PO_4_ particles. The EDS mapping (Figure 2e) shows that the Ag, Y, P, Fe, Co, and O elements are all distributed in Ag_3_PO_4_/CoFe_1.95_Y_0.05_O_4_–0.2. These findings suggest that the CoFe_1.95_Y_0.05_O_4_ nanoparticles can effectively regulate the formation and growth of the Ag_3_PO_4_ particles, resulting in the formation of uniformly coupled Ag_3_PO_4_/CoFe_1.95_Y_0.05_O_4_ composites. The smaller particle size of Ag_3_PO_4_ will result in a larger surface area, thus facilitating the utilization of the incident irradiation energy and photocatalytic reactions through effectively separating the electrons and holes [15].

### 3.2. Optical Properties of the Samples

**Figure 3:**
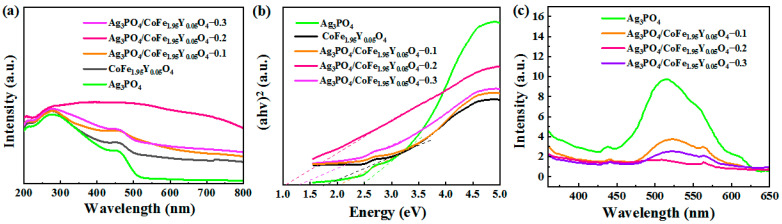
(**a**) DRS spectra and (**b**) the calculated band gap energies diagram of Ag_3_PO_4_, CoFe_1.95_Y_0.05_O_4_, and Ag_3_PO_4_/CoFe_1.95_Y_0.05_O_4_ nanocomposites; (**c**) PL curves of Ag_3_PO_4_ and Ag_3_PO_4_/CoFe_1.95_Y_0.05_O_4_ nanocomposites.

It is worth noting that Ag_3_PO_4_ has the main ability of absorbing light in the UV region, but with minimal absorption of incident light when the wavelength exceeds 500 nm [14]. To explore the light absorption capacity of the Ag_3_PO_4_/CoFe_1.95_Y_0.05_O_4_ nanocomposites, Diffuse Reflectance Spectrum (DRS) tests are conducted and are presented in Figure 3a, and it can be seen that pure Ag_3_PO_4_ has the ability to absorb light in both the UV and visible regions, with a majority of the absorption occurring in the UV region. When the wavelength of the incident light exceeds 500 nm, there is almost no absorption anymore. Comparatively, the Ag_3_PO_4_/CoFe_1.95_Y_0.05_O_4_ nanocomposites exhibit a high absorption efficiency in both the UV and visible regions, particularly in the visible region, where the absorption intensity is significantly amplified.

Figure 3b shows the Kubelka–Munk function band gap energy (Eg) plot, indicating that the band gap energies of all Ag_3_PO_4_/CoFe_1.95_Y_0.05_O_4_ nanocomposites are lower compared to pure Ag_3_PO_4_ (2.5 eV). In fact, the band gap energies of Ag_3_PO_4_/CoFe_1.95_Y_0.05_O_4_ nanocomposites can reach 1.2 eV along with the increase in CoFe_1.95_Y_0.05_O_4_. This demonstrates that coupling with CoFe_1.95_Y_0.05_O_4_ significantly improves the light absorption ability of Ag_3_PO_4_, and simultaneously reduces its band gap. Furthermore, the conduction band (CB) and valence band (VB) energy levels can be given by equations of E_CB_ = X – Ee − 0.5Eg and E_VB_ = X − Ee + 0.5Eg, respectively, where the value of Ee is 4.5 eV and X is absolute electronegativity, which is 4.97 for Ag_3_PO_4_ and 5.83 for CoFe_1.95_Y_0.05_O_4_. Therefore, the CB is calculated as 0.21 and 0.73 eV, while the VB is calculated as 2.71 and 0.93 eV for Ag_3_PO_4_ and CoFe_1.95_Y_0.05_O_4_, respectively.

To investigate the photo-separation efficiency of electron–hole pairs in the prepared samples, we conduct Photoluminescence Spectra (PL) tests on Ag_3_PO_4_/CoFe_1.95_Y_0.05_O_4_ nanocomposites. As shown in Figure 3c, the pure Ag_3_PO_4_ displays a prominent emission peak at 535 nm, which results from the complexation of excited state electrons in the conduction band and holes in the valence band. Comparatively, the emission intensity of the Ag_3_PO_4_/CoFe_1.95_Y_0.05_O_4_ nanocomposites is found to be significantly reduced. This is due to the heterojunction structure of the Ag_3_PO_4_/CoFe_1.95_Y_0.05_O_4_ nanocomposites, which suppresses the complexation of the electrons and holes, thereby enhancing the photocatalytic efficiency [16].

### 3.3. Photocatalytic Properties

**Figure 4:**
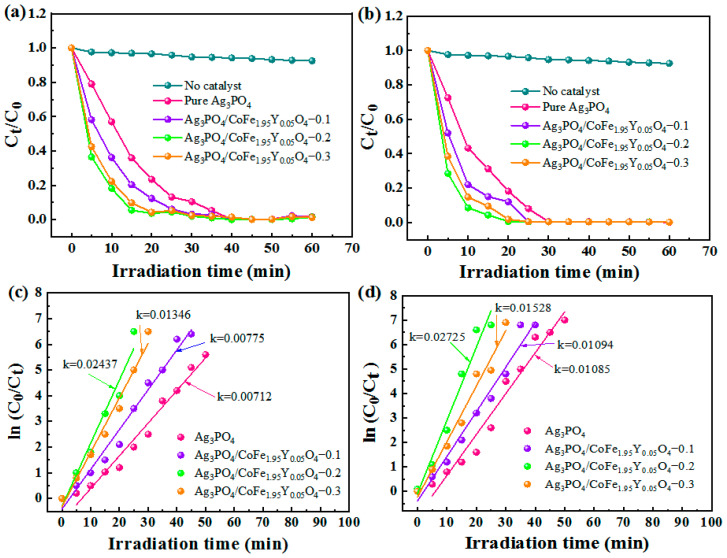
Degradation curves for (**a**) RhB and (**b**) MB. The corresponding first-order kinetics of (**c**) RhB and (**d**) MB of the Ag_3_PO_4_/CoFe_1.95_Y_0.05_O_4_ nanocomposites.

The photodegradation performance of the Ag_3_PO_4_/CoFe_1.95_Y_0.05_O_4_ nanocomposites is analyzed and presented in Figure 4. The results show that all the samples exhibit excellent degradation behaviors for both MB and RhB (Figure 4a,b), The RhB and MB can be degraded within 40 and 30 min, respectively. Specifically, the Ag_3_PO_4_/CoFe_1.95_Y_0.05_O_4_ nanocomposites demonstrate a higher degradation efficiency compared to pure Ag_3_PO_4_. The degradation efficiency of Ag_3_PO_4_/CoFe_1.95_Y_0.05_O_4_–0.2 is found to be higher than those of the Ag_3_PO_4_/CoFe_1.95_Y_0.05_O_4_–0.1 and Ag_3_PO_4_/CoFe_1.95_Y_0.05_O_4_–0.3 samples. This suggests that the mass ratio of Ag_3_PO_4_ to CoFe_1.95_Y_0.05_O_4_ is important to obtain optimized performance. An overabundance of CoFe_1.95_Y_0.05_O_4_ may actually decrease the degradation rate because they may cover the Ag_3_PO_4_ surface and block light, ultimately leading to lower degradation efficiency. The photocatalytic degradation efficiency of the composite materials is competitive to other reported photocatalysts (Table 1) [24,25,26,27,28,29,30].

The primary reaction kinetics equation is satisfied due to the low concentrations of the dyes [2,10]. Figure 4c,d show the ln(C_0_/C_t_) versus light time curves of the RhB and MB, respectively. It is shown that Ag_3_PO_4_/CoFe_1.95_Y_0.05_O_4_–0.2 has the fastest degradation rate among all samples, with apparent reaction rate constants of 0.02437 and 0.02725 min^−^^1^ for RhB and MB, respectively, while the reaction rate constants of Ag_3_PO_4_/CoFe_1.95_Y_0.05_O_4_–0.3, Ag_3_PO_4_/CoFe_1.95_Y_0.05_O_4_–0.1, and Ag_3_PO_4_ are 0.01346, 0.00775, and 0.00712 min^−^^1^, respectively. The reaction rate constants of Ag_3_PO_4_/CoFe_1.95_Y_0.05_O_4_–0.2 are 1.81, 3.14, and 3.42 times higher than those of Ag_3_PO_4_/CoFe_1.95_Y_0.05_O_4_–0.3, Ag_3_PO_4/_CoFe_1.95_Y_0.05_O_4_–0.1, and Ag_3_PO_4_, respectively.

**Figure 5:**
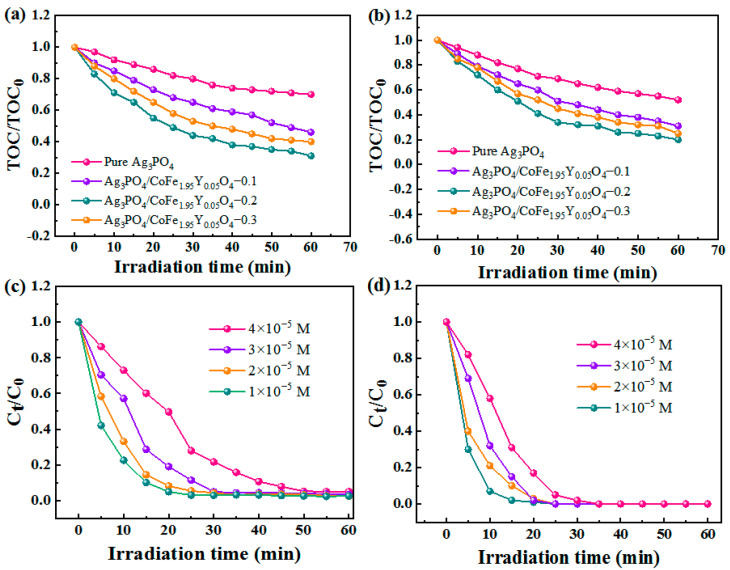
TOC removal during the photodegradation process for (**a**) MB and (**b**) RhB. The concentration effects of (**c**) RhB and (**d**) MB on the photodegradation efficiency of the Ag_3_PO_4_/CoFe_1.95_Y_0.05_O_4_–0.2.

Figure 5a,b display the TOC changes during the degradation of MB and RhB. Although the catalyst successfully decolorizes all dye solutions, the desired level of TOC removal is not achieved. This suggests that not all dye molecules can be catalyzed to inorganic molecules, and the dye concentration has an impact on the degradation efficiency. It is indicated that the Ag_3_PO_4_/CoFe_1.95_Y_0.05_O_4_–0.2 nanocomposite exhibits the most effective photocatalytic performance. The TOC removal rates of Ag_3_PO_4_/CoFe_1.95_Y_0.05_O_4_–0.2 are approximately 0.35 and 0.20 for MB and RhB, respectively, after 60 min of degradation. Figure 5c–d display the degradation efficiencies of Ag_3_PO_4_/CoFe_1.95_Y_0.05_O_4_–0.2 for various concentrations of MB and RhB dyes. The initial degradation during the first 5 min is slightly slower under higher dye concentrations. The main reason is that the dark color of the dye shields the light. When the dye concentrations reduce to 4 × 10^−^^5^ mol·L^−^^1^, the photocatalyst is able to degrade all of the RhB dye within 30 min and completely degrade the MB dye within 50 min.

### 3.4. Enhancement Mechanism and Stability

**Figure 6:**
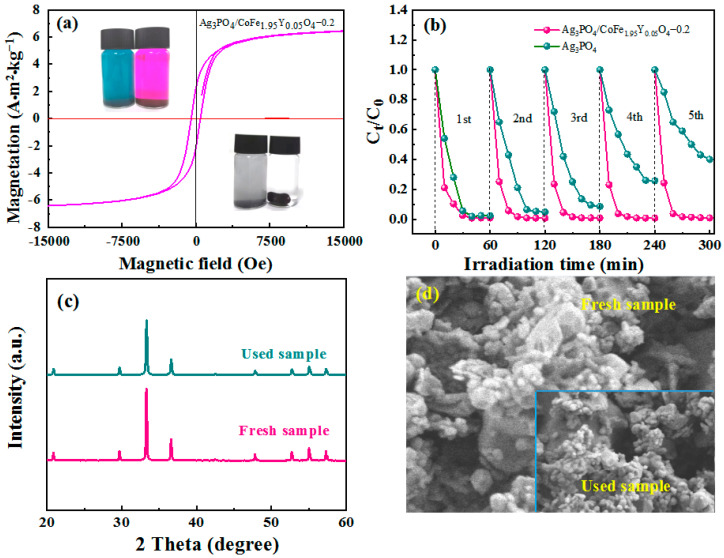
(**a**) Magnetization curve of Ag_3_PO_4_/CoFe_1.95_Y_0.05_O_4_–0.2 after five degradation cycles for MB (inset shows the visual pictures of the solution after photocatalytic degradation and magnetic field recovery); (**b**) five degradation cycling curves of the photocatalysts for MB; (**c**) XRD patterns and (**d**) SEM images of Ag_3_PO_4_/CoFe_1.95_Y_0.05_O_4_–0.2 before and after five degradation cycles for MB.

As shown in Figure 6a, the Ag_3_PO_4_/CoFe_1.95_Y_0.05_O_4_–0.2 catalyst exhibits a saturation magnetization intensity of 6.48 A·m^2^·kg^−^^1^. As a result, the catalyst can be conveniently separated from the dye using a magnet once the degradation process is finished, which is a benefit for the recycling and reuse of the photocatalyst, effectively preventing any secondary pollution that may result from the addition of the photocatalyst [20,23]. In this study, MB is selected as the target dye for five cycles of catalytic degradation. The results of the degradation process are illustrated in Figure 6b. It can be seen that there is almost no degradation decay of the Ag_3_PO_4_/CoFe_1.95_Y_0.05_O_4_–0.2 nanocomposite compared to Ag_3_PO_4_. Furthermore, the XRD patterns (Figure 6c) and SEM images (Figure 6d) show that the phase and morphology of the photocatalyst remain unchanged after five degradation cycles. This suggests that the crystal structure of the composite is stable, and the photocatalyst exhibits both efficient photocatalytic performance and good cycling stability during the photodegradation process.

**Figure 7:**
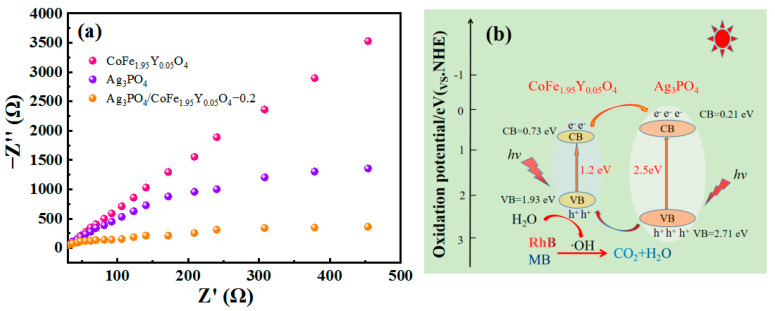
(**a**) EIS spectra of the samples; (**b**) schematic illustration of the degradation mechanism.

The Nyquist diagram (Figure 7a) demonstrates that Ag_3_PO_4_/CoFe_1.95_Y_0.05_O_4_–0.2 has a smaller arc radius compared to Ag_3_PO_4_ and CoFe_1.95_Y_0.05_O_4_, suggesting that the coupling structure of Ag_3_PO_4_/CoFe_1.95_Y_0.05_O_4_–0.2 is able to quickly transport and effectively separate photogenerated carriers. Based on the above discussion, a photocatalyst degradation mechanism is proposed as shown in Figure 7b; when subjected to continuous light irradiation, the electrons in Ag_3_PO_4_ and CoFe_1.95_Y_0.05_O_4_ become excited from the valence band to the conduction band, leading to the creation of valence band holes. These holes then react with H_2_O, generating ·OH radicals that degrade the dye. And the electrons migrate to the Ag_3_PO_4_ layer, where they participate in the photocatalytic reaction. This mechanism enhances the separation of photogenerated electron–hole pairs and improves the photocatalytic activity of the Ag_3_PO_4_/CoFe_1.95_Y_0.05_O_4_ nanocomposite. The conduction band potential of CoFe_1.95_Y_0.05_O_4_ is more negative than that of Ag_3_PO_4_, resulting in a continuous transfer of excited electrons to the conduction band of Ag_3_PO_4_. Additionally, the valence band of Ag_3_PO_4_ has a higher energy level than that of CoFe_1.95_Y_0.05_O_4_, leading to a continuous transfer of holes from the valence band of Ag_3_PO_4_ to that of CoFe_1.95_Y_0.05_O_4_. The transferred vacancies react with H_2_O to produce the highly reactive product of ·OH radical. This reaction is enhanced by the presence of CoFe_1.95_Y_0.05_O_4_, which can effectively prevent the recombination of the electrons and holes. As a result, the Ag_3_PO_4_/CoFe_1.95_Y_0.05_O_4_ nanocomposites exhibit a higher photodegradation performance.

## 4. Conclusions

In this study, Ag_3_PO_4_/CoFe_1.95_Y_0.05_O_4_ magnetic nanocomposites are prepared and used as photocatalysts against the degradation of MB and RhB dyes. It is observed that the particle size of the Ag_3_PO_4_ in nanocomposites can be effectively reduced compared to the pure Ag_3_PO_4_. Furthermore, the addition of CoFe_1.95_Y_0.05_O_4_ can enhance the light absorption efficiency of Ag_3_PO_4_, resulting in a narrower forbidden band width, allowing the effective degradation of dyes within 30 min at concentrations of up to 4 × 10^−^^5^ mol·L^−^^1^. The degradation cycling tests show that the Ag_3_PO_4_/CoFe_1.95_Y_0.05_O_4_ nanocomposite demonstrates superior photocatalytic stability. On the other hand, the Ag_3_PO_4_/CoFe_1.95_Y_0.05_O_4_ nanocomposite exhibits exceptional magnetic properties, which enables the recycling and reuse of the photocatalyst. This study involves the development of a new photocatalyst to achieve efficient, stable degradation and magnetic separation, providing a new way for photocatalytic degradation of organic pollutants in wastewater.

## Figures and Tables

**Table 1:** Comparison of the photocatalytic properties between Ag_3_PO_4_/CoFe_1.95_Y_0.05_O_4_–0.2 and other reported photocatalysts.

Catalysts	Pollutants	Degradation Efficiency (%)	Irradiation Time (min)	Ref.
S-NaTaO_3_/biochar	RhB	99.6	90	[24]
BaTiO_3_/TiO_2_	MB	100	180	[25]
LiNb_3_O_8_	MB	96.9	100	[26]
BiMnVO_5_	MB	98	240	[28]
CdS	MB	97.8	30	[29]
MoS_2_-ZnO	MB	97	20	[30]
Ag_3_PO_4_/CoFe_1.95_Y_0.05_O_4_–0.2	MB	98	30	Us

## Data Availability

Not applicable.
